# Co-Occurrence of Exogenous and Endogenous Cushing's Syndromes—Dilemma in Diagnosis

**DOI:** 10.1155/2019/2986312

**Published:** 2019-10-13

**Authors:** Chin Voon Tong, Subashini Rajoo

**Affiliations:** ^1^Department of Medicine, Malacca Hospital, 75400, Malaysia; ^2^Department of Medicine, Kuala Lumpur Hospital, 50586, Malaysia

## Abstract

Approach to patients who manifest with features of Cushing's syndrome often begin with exclusion of exposure to excessive exogenous source of glucocorticoids (GC). Most guidelines advocate no further assessment if excessive exogenous GC use is present. We present a case of a 66-year-old lady who was noted to have typical features of Cushing's syndrome. As she gave a very clear history of ingesting exogenous GC for a year, no further work up was undertaken. Despite cessation of GC for a year, she continued to have thin skin and easy bruising. Upon admission for hypertensive emergency, her clinician took note of her changes and investigated her for endogenous Cushing's syndrome. Her cortisol post overnight dexamethasone suppression test was 707 nmol/l. Post low dose dexamethasone suppression test yielded a cortisol of 1133.2 nmol/l. 24 hours urine cortisol was 432.2 nmol/l. Plasma ACTH was 1.1 pmol/l, indicating an ACTH independent Cushing's syndrome. We proceeded with Computed tomography scan (CT scan) of adrenals which revealed a right adrenal adenoma measuring 4.4 × 3.4 × 4.0 cm. Right retroperiteneoscopic adrenalectomy was done. Histopathology examination was consistent with adrenal cortical adenoma with foci of myelolipoma. Post adrenalectomy she developed hypocortisolism secondary to contralateral adrenal suppression which lasted up to the present date. Her cutaneous and musculoskeletal manifestations improved substantially. Co-occurrence of endogenous and exogenous Cushing's syndromes is uncommon but should be considered in patients whose Cushingnoid features do not resolve after cessation of exogenous GC.

## 1. Introduction

While endogenous Cushing's syndrome is rare, with reported incidence of 2–3 per 1 million inhabitants per year [1], the actual incidence of exogenous or iatrogenic Cushing's syndrome is not known. The source of exogenous GC also varies from those being used in clinical setting to unknowing ingestion from traditional treatments. A local study found that 82% of older adults consume traditional medications. Samples of traditional medications used in this study were analysed and more than half (61.8%) contained steroids [2]. Traditional Chinese medications (TCM) are also popular among local population, including non-Chinese. While not all herbal TCM products contain steroids, adulteration of these products with exogenous steroids has been detected [3]. We report herein a patient who had both endogenous and exogenous Cushing's syndromes which contributed to a delay and dilemma in diagnosis.

## 2. Materials and Methods

Patient's file was retrieved and reviewed retrospectively.

## 3. Results

### 3.1. History

Our patient is a 66-year-old housewife whose daughter is a dentist and was able to give accurate medical history. She was noted to be Cushingnoid in 2009 by her attending physician. She had thin skin with easy bruising, truncal obesity, and proximal myopathy. At that time, she gave a clear history of ingesting TCM which was known to contain GC for a year. The medications were taken for general well-being. She was seen at another hospital and we were not able to trace her baseline cortisol taken then. She was advised to, and subsequently stopped taking it. After cessation of the exogenous GC, there was slight improvement in her Cushing's features, but they became more noticeable again in 2017. She then developed hypertension in 2010, diabetes mellitus in 2016, and had an osteoporotic fracture in 2011. During an admission for hypertensive crisis in 2017, a physician noticed her features and worked her up. During this admission, she had severe hypokalemia as well as hyponatremia. She was, however, on Indapamide for her hypertension prior to that.

### 3.2. Investigations

Her hormonal profile was consistent with ACTH independent Cushing's syndrome. Her results are summarized in Table 1.

**Figure 1:**
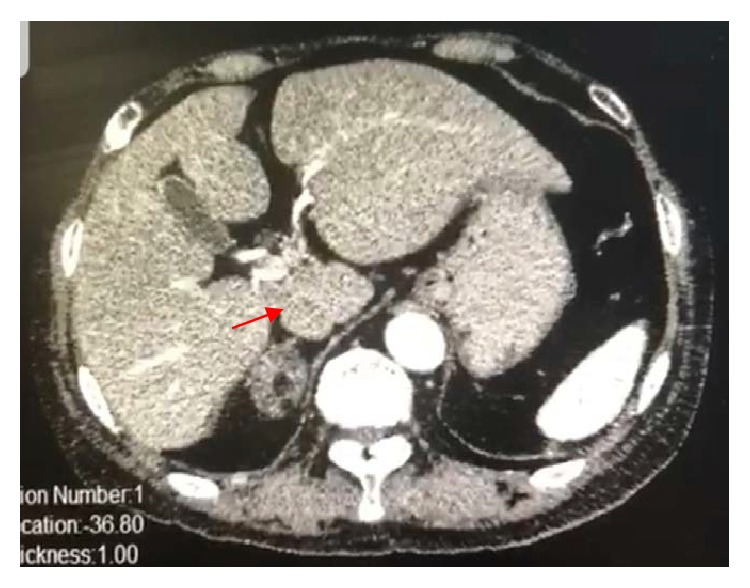
CT scan adrenal showing right adrenal adenoma.

Computed tomography (CT) scan of adrenal showed a right adrenal adenoma measuring 4.4 × 3.4 × 4.0 cm. Contralateral adrenal was normal (Figure 1). 24 hours metanephrines were not elevated.

### 3.3. Management

She underwent right retroperiteneoscopic adrenalectomy. Prior to surgery, she was given ketoconazole to control her cortisol excess. She required four anti hypertensives and one glucose lowering medications. Intraoperatively, a right adrenal adenoma measuring 4 × 4 cm was found. Post surgery, she required inotropic support for 24 hours despite being given hydrocortisone cover peri-operatively. Histopathology examination was consistent with adrenal cortical adenoma with foci of myelolipoma. She is currently into her 13^th^ month post adrenalectomy. Her cutaneous manifestations have improved markedly. She now requires a single anti-hypertensive but is euglycemic without any glucose lowering therapies. Her cortisol remained suppressed with a pre dose cortisol of <13 nmol/l. She is now taking a physiological dose of oral hydrocortisone of 10 mg in the morning and 5 mg in the afternoon.

## 4. Discussion

Co-occurrence of both endogenous and exogenous Cushing's syndromes is rare, though its true incidence is not known. Both share similar clinical presentations. Some authors have reported certain differences in the two. Exogenous Cushing's syndrome tends to have more striking clinical features, relatively less hypertension and hypokalemia as well as increased incidence of glaucoma [4]. However, in our experience, it is often difficult to differentiate both based on clinical features. In our local setting, a thorough and repeated history taking is important to establish intake of exogenous GC, especially from alternative sources. Patients are often reluctant to reveal this. The exact dose or amount of corticosteroids taken that will lead to iatrogenic Cushing's syndrome is not known as multiple factors such as formulations used, pharmacokinetics, biologic potency, affinity for glucocorticoid activity and duration of action play a role in causing Cushingnoid features [4].

Prolonged exposure to exogenous GC can potentially lead to secondary adrenal insufficiency caused by suppression of the hypothalamic–pituitary–adrenal (HPA) axis. The amount, duration and mode of delivery of exogenous GC that causes secondary adrenal suppression varies between individual and is difficult to predict. Schlaghecke et al. who studied more than 200 patients receiving daily GC therapies concluded that pituitary-adrenal function in these patients cannot be accurately estimated from the dose of GC, the duration of therapy, or the basal cortisol concentration [5]. In terms of mode of delivery, even inhaled GC which were thought to be safe in the past, and unusual mode such as rectal GC have also been reported to cause adrenal crisis upon its withdrawal [6, 7].

**Figure 2:**
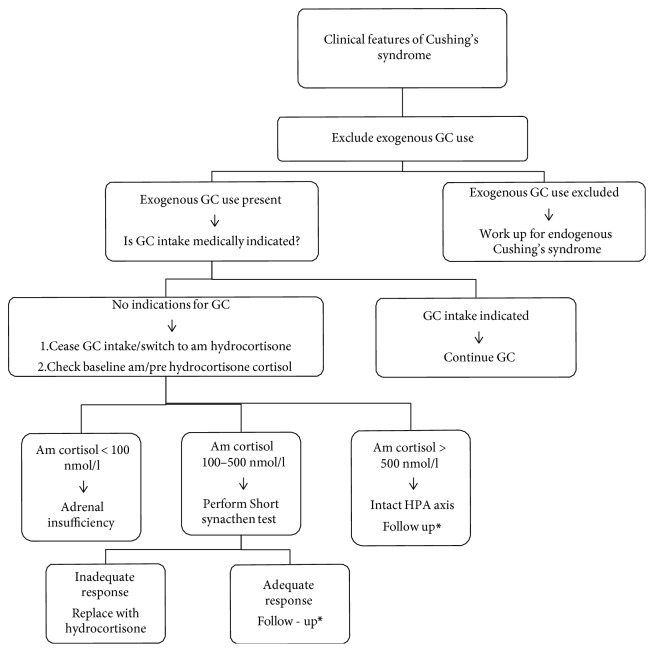
Algorithm for workup of a patient with exogenous Cushing's syndrome. ^∗^Glucocorticoids tapered and off. Patients are followed up and assessed for resolution of Cushingnoid features. If features persist, to consider concurrent endogenous Cushing's syndrome.

Work up for a patient who presents with Cushing's syndrome should include assessment for adrenal insufficiency in those whose cause is deemed to be from an exogenous source. Bearing in mind that both can coexist, further follow up is still necessary for patients who are thought to have exogenous Cushing's syndrome and still have intact HPA axis. Our suggested approach to a patient presenting with features of Cushing's syndrome is depicted in Figure 2.

If exogenous GC use has been excluded, the work up is done for endogenous Cushing's syndrome [1]. In the event that there is use of exogenous GC, the use is continued if medically indicated. However if there is no clear indication, the GC is either ceased or switched to a morning dose of hydrocortisone. A prehydrocortisone morning cortisol is then measured. A level of <100 nmol/l indicates adrenal insufficiency and replacement is initiated. If the am cortisol ranges between 100 and 500 nmol/l, a short synacthen test is performed. Am cortisol of >500 nmol/l is an indication of intact HPA axis [8]. Clinicians should also be aware that GC like dexamethasone suppresses cortisol levels and others such as hydrocortisone are measurable in cortisol assays. We suggest further follow up for patients with intact HPA axis to assess for improvement of Cushingnoid features after cessation of GC. Persistence of symptoms warrants a further workup for concurrent endogenous cause.

## 5. Conclusion

In conclusion, co-occurrence of endogenous and exogenous Cushing's syndromes is uncommon but should be considered in patients whose Cushingnoid features do not resolve after cessation of exogenous glucocorticoids.

## Figures and Tables

**Table 1:** Hormonal work up for Cushing's syndrome.

Parameters	Results
Morning cortisol (nmol/l)	841.2
Cortisol post overnight dexamethasone suppression test (nmol/l)	707.3
Cortisol post low dose dexamethasone suppression test (nmol/l)	1133.2
Plasma adrenocorticotropic hormone (pmol/l)	1.1
24 hours urine cortisol (nmol/L)	432.2
